# The ANT Home Care Model in Palliative and End-of-Life Care. An Investigation on Family Caregivers’ Satisfaction with the Services Provided

**DOI:** 10.37825/2239-9747.1022

**Published:** 2020-10-01

**Authors:** V Zavagli, M Raccichini, R Ostan, L Franchini, A Bonazzi, S Varani, R Pannuti

**Affiliations:** 1 ANT Italia Foundation, Bologna, Italy

**Keywords:** family caregivers, palliative care, cancer, home care model, healthy ageing, end-of-life care

## Abstract

The World Health Organization plan for a *Decade of Healthy Ageing 2020–2030* has established some priorities in the field of palliative and end-of-life care. It states that “people require non-discriminatory access to good-quality palliative and end-of-life care” and recommends the “implementation of strategies for the provision of information, training, respite and support for informal caregivers”. The priorities described are in line with the home care services that National Tumor Assistance (ANT) Foundation has been providing in Italy. This 5-years investigation was designed to measure caregivers’ satisfaction and determine what types of support services are associated with greater satisfaction. 5.441 family caregivers filled out autonomously a 6-item questionnaire at the end of home care assistance, focusing on the level of satisfaction with the social and health services received. The overall data indicate a high satisfaction rate for the home care assistance received. In particular, participants rate positively the assistance provided by healthcare professionals (physicians, nurses and psychologists). The most appreciated aspects of assistance are those ensuring a global management of patients and their families, whereas an area of deficiency emerged was the continuity of care, suggesting the importance to implement the networks between the health care facilities and home care services. The present investigation constitutes a mean to highlight the aspects associated with greater satisfaction and the ones perceived as less satisfactory by caregivers. Moreover, this research constitutes a crucial instrument to improve home care assistance provided by ANT ensuring the best quality of life for both patients and their families.

## I. INTRODUCTION

The World Health Organization (WHO) has defined *Healthy Ageing* as “the process of developing and maintaining the functional ability that enables well-being in older age” [1–3]. This definition implies a life-course approach: actions to improve healthy ageing should be taken at all ages, with particular attention to the needs of people at critical life stages. Moreover, actions should be developed at multiple levels and in multiple areas in order to prevent disease, promote health, maintain physical and mental capacities and build functional abilities.

The WHO plan for a *Decade of Healthy Ageing 2020*–*2030* is “an opportunity for ten years of concerted, catalytic and collaborative action” on Healthy Ageing. The guiding principle is that “no one will be left behind and that every human being will have the opportunity to fulfill their potential in dignity and equality” [4].

Regarding the concrete actions to reach the objectives of the plan, the WHO has established some main priorities. Among these, it emerges that “people require non-discriminatory access to good-quality palliative and end-of-life care”. It is also recommended “the implementation of strategies for the provision of information, training, respite and support for informal caregivers”. In fact, informal caregivers are a vulnerable group: considerable research on caregiving portrays it as a stressful and burdensome experience and many studies show that family carers experience severe strain, with serious negative consequences on their physical and mental health [5–14].

The priorities described are in line with the home care support services that National Tumor Assistance (ANT) Foundation has been providing in Italy for over 40 years. ANT is an Italian no-profit Foundation that provides free medical, nursing, psychological and social home oncological assistance in 11 Italian regions since 1985. According to the biopsychosocial model, ANT provides support not only focusing on the biological determinants of disease, but understanding patients’ subjective experiences and tailoring care to the physical, emotional, and social well-being of the patients as well as their families. In fact, cancer affects the whole family, not only the person with the disease, going to influence cognitive, emotional, relational, and spiritual aspects [15, 16].

From an integrated care perspective, ANT multidisciplinary team of health care professionals work together to help patients and their families to live with dignity, despite the impact of the disease. In fact, the support for family caregivers is a core aspect of palliative care provision. It is recognized that their needs must be assessed and addressed appropriately, as their physical and mental well-being affects the quality of care they provide to the patients also. Moreover, recent studies [17–20] show that home care service impacts positively on caregivers’ health and burden of caring when facing the end-of-life and it is also shown that it improves bereavement outcomes [21].

ANT model provides a multidisciplinary approach to cancer patients, who are taking in charge by a health care professional team from a holistic perspective. Medical and nursing services are active 24/7 all over the assistance period, until the decease. Psychological support is also provided both for patients and carers during the ANT assistance and even after the patient death, for the mourning process. In particular, ANT Psychological Service for family caregivers offers individual and group therapy, emotional support, counseling, and psycho-education. Furthermore, caregivers can participate in a training course aimed at empowering and assisting them with strategies, techniques and useful tips. A Caregiving Guide containing recommendations, tools and resources is available and may help carers to better support their relatives.

Families assisted by ANT can also activate any social services to mitigate the concrete difficulties due to the cancer disease as the provision of drugs not reimbursable by public health system, free transport for day hospital admissions, provision of medical aids (as orthopedic beds, infusion pumps, wheelchairs etc.), cleaning of bed sheets and blankets including collection and delivery.

Given the importance and the interest of the *Decade of Healthy Ageing* priorities described above, the aim of this paper is to share our experiences and approaches in palliative and end-of-life care. In particular, this investigation was designed to measure caregivers’ satisfaction and determines what types of support services are associated with greater satisfaction. In fact, caregivers’ perceptions and evaluations of the assistance provided is a core point in establishing and optimizing these services and ensuring their success.

## II. METHODOLOGY

**Figure 1:**
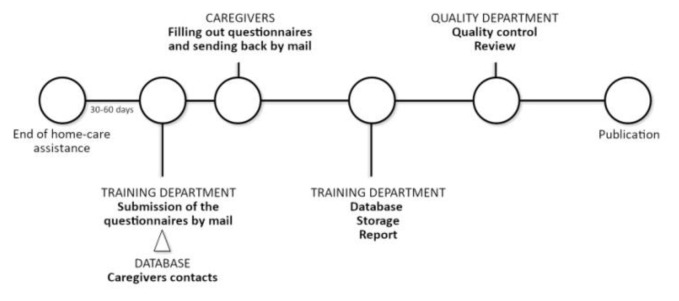
Timetable and procedure of the investigation.

Participants were enrolled in Italy among the family caregivers of the patients assisted by the ANT Foundation through its 23 oncological hospitals at home, over a period of 5 years, from 2014 to 2018. Data were collected from 5.441 family caregivers through a postal survey method. Participants filled out autonomously a 6-item questionnaire focusing on level of caregiver’s satisfaction with the social and health services received. They completed the questionnaire at the end of home-care assistance provided by ANT Foundation to their sick relatives, according to the timetable and procedure presented in Figure 1.

A postal survey method was chosen because of the availability of caregivers’ contact addresses, which would obviate any potential selection bias. This technique has the advantage that subjects feel free to express themselves openly, compared with face-to-face interviews. On the other hand, it could have a negative effect on the completeness of the data collected.

The questionnaire uses a 7-point Likert response scale from “not at all satisfied” to “very satisfied”. Questions 1 to 5 require an evaluation of the quality of the intervention by the health care professionals (physician, nurse, and psychologist if activated), family services and the overall assistance received. Question 6 asks to indicate which aspects of the assistance were more satisfied among the following (maximum 3 answers): Professionalism and speed of intervention; Continuity of care; Availability and humanity; Supply of medicines and devices; ANT assistance activation (c/o Reception office); Consultations and diagnostic tests at home; Facility of access to the Psychology service; Information on ANT services; Social Assistance by volunteers (if any); Social services.

In addition, socio-demographic data were retrieved (sex, age, marital status, education level).

## III. RESULTS

All analyses were conducted using SPSS 24.0 for Windows.

For the total sample of family caregivers, frequencies, mean and standard deviation scores for satisfaction’s questionnaire were calculated.

From 2014 to 2018 ANT Foundation sent by post 21.774 paper questionnaires to potential participants caregivers. 5.441 of those returned filled (25.4% of the total).

A summary of the characteristics of the subjects who took part in the investigation is presented in Table 1. The socio-demographic data of our sample confirm the characteristics already observed in the literature about cancer caregivers. In fact, this role is played mostly by women (67–78%) with an average age of 41–60 years.

**Figure 2:**
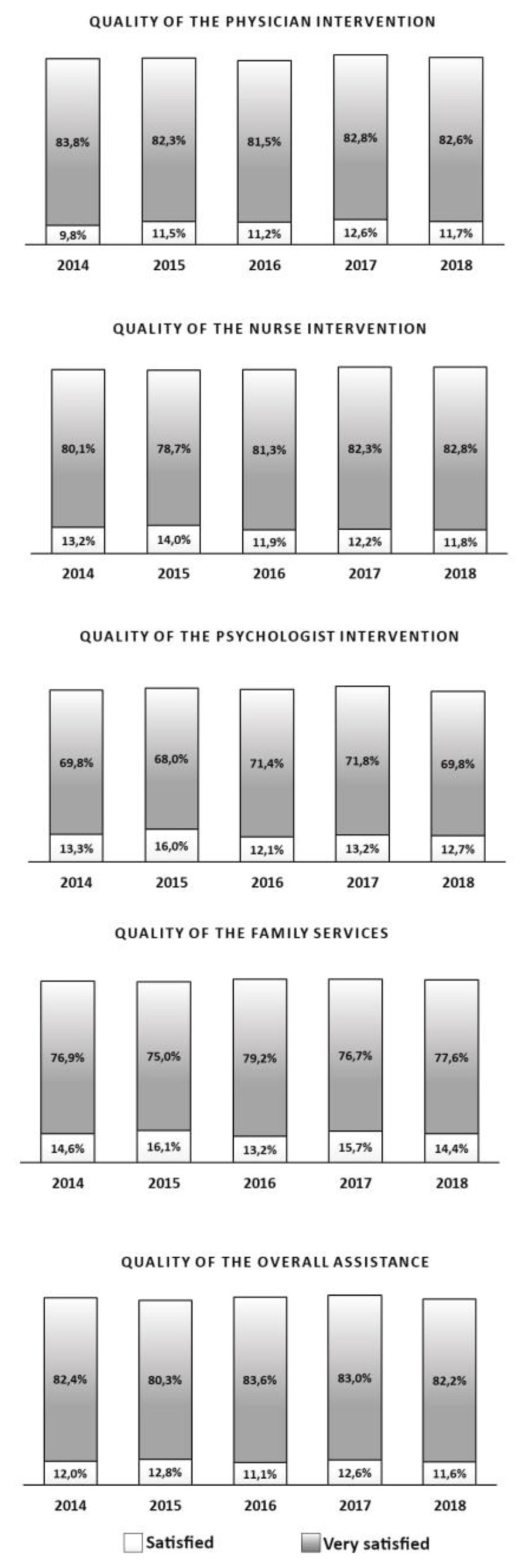


The overall data for the 5 years indicates a high satisfaction rate with regards to the home care assistance received: 94.3% of respondents indicated that they were “satisfied” or “very satisfied”. Comparing the data from northern, central and southern Italy, the percentages of satisfaction show that there are no significant differences among the geographical areas with values of 95.4%, 96.5% 96.5% and 91.7% of “satisfied” or “very satisfied” participants respectively. The distribution of the caregivers’ answers is shown in Figure 2.

The participants reported a high level of satisfaction regarding the quality of the intervention provided by the physician, ranging from 81.5% to 83.8%, during the five years taken into consideration. The same level of satisfaction is reported as regards the quality of intervention provided by nurses and psychologists. Respectively, the percentage of satisfaction range from 78.7% to 82.8% and from 68.0% to 71.8%. As concern the intervention of family services, the level of satisfaction reported by caregiver ranged from 75.0% to 79.2%.

In general, to question 5, referring to overall assistance provided by ANT, the participants reported a high level of satisfaction, with percentages from 80.3% to 83.6%.

**Figure 3:**
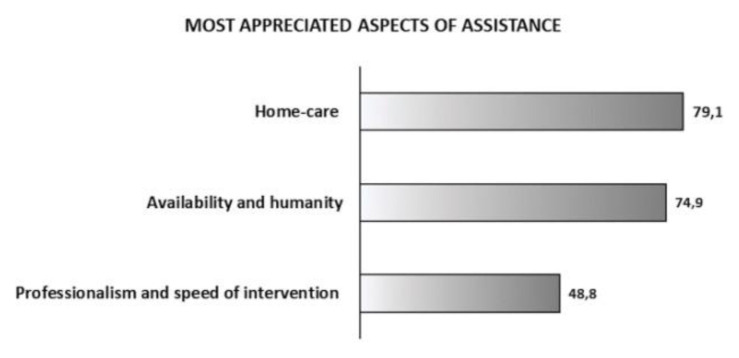
Caregivers’ most appreciated aspects of assistance.

Finally, regarding to the most appreciated aspects of assistance, the caregivers indicated in most cases those that ensure a global management of the patient and his family. In particular, as figure 3 shows, the aspects of service considered more satisfying are: home care (79.1%), availability and humanity (74.9%), and professionalism and speed of intervention (48.8%). On the contrary, caregivers expressed low levels of satisfaction with the continuity of care (33.7%).

## IV. DISCUSSION

The measurement of customer satisfaction has become widespread in both healthcare and social care services. It constitutes a fundamental instrument for providing interventions that ensure the best quality of life for both the patients and their families.

The aim of the present investigation was to measure caregivers’ satisfaction in order to improve home support assistance provided by the ANT Foundation. Therefore, it was essential to identify both what types of support services were associated with greater satisfaction and what caregivers perceive as less satisfactory.

The results show an overall high level of satisfaction, which is homogeneous throughout the Italian Regions where ANT is present and remains constant over the 5 years of evaluation. The vast majority of caregivers is satisfied with the home care assistance received. In particular, they rate positively the assistance provided by healthcare professionals (physicians, nurses and psychologists).

According to previous studies [22, 23], they express satisfaction for all the aspects of assistance that ensure a global management of the patients and their families. In fact, large proportions of caregivers evaluate positively the 24/7 availability of the service, professionalism, sensitivity and flexibility in meeting patients’ and families’ needs, and practical adjustments to enable care at home.

These findings are in line with those reported in the literature on the topic: in fact, in congruence with our results, some studies [24–26] have found that the most common reason for dissatisfaction is the caregivers’ perception of having no influence on the services offered.

It is already known from the literature that the shift of care from hospitals and hospices to home can be beneficial for both patients and their family caregivers as long as integrated teamwork, management of pain and physical symptoms, holistic care, compassionate and skilled providers, timely and responsive care, and patient and family preparedness are guaranteed [27]. Therefore, our results are consistent with previous findings: home palliative care teams improve patients’ and caregivers’ experience of security by providing competent care, being present, and caring for the family as a unit [28, 29].

Family caregivers are an invaluable part of healthcare teams and improving the relationship between them and professionals can be important for all parties involved. First of all, healthcare professionals can rely on the knowledge of family caregivers; secondly, family caregivers can feel involved and enhance their confidence in caring for the patients; finally, patients can receive better assistance since formal and informal carers are better aligned to each other.

Moreover, as the majority of family caregivers is female and literature has found that female caregivers show to have more distress and a poorer quality of life than males, it is important to pay attention in providing appropriate support and training to this kind of population by addressing inequality and making caregiving less burdensome [30, 31].

Although the results show that most family caregivers are satisfied with the services provided by ANT Foundation, there are still areas of deficiency, particularly in the continuity of care. It suggests the importance to implement the networks between the different care settings, mostly between health care facilities and the home care services.

The present investigation has practical and operative implications for advancing healthcare research and practice. The findings can have a relevance to good clinical experience, suggesting that practical strategies for providing home care services should be developed through a concrete assessment of the family dynamics and family caregivers’ needs, that can be culturally determined. At the same time, such dynamics and needs of families in-home care assistance should be continuously monitored with constant detections.

## V. CONCLUSION

We strongly believe that the exchange of experiences and approaches in palliative and end-of-life care is a critical step for reducing gaps in active and healthy ageing worldwide, with a life-course approach.

This investigation has corroborated the six essential elements of quality palliative homecare identified in the literature: (I) Integrated teamwork; (II) Management of pain and physical symptoms; (III) Holistic care; (IV) Caring, compassionate, and skilled providers; (V) Timely and responsive care; and (VI) Patient and family preparedness [27]. These components are also mentioned among the *Decade of Healthy Ageing* priorities and can constitute a basis for develop and publish clinical practice guidelines for quality home-based palliative care. Furthermore, investigations of this kind can urge legislators to recognize the caregivers’ figure. In fact, there is not yet a common European law that protects and improves the role and status of the caregivers and the different countries has a different cultural approach to the issue.

## Figures and Tables

**Table 1:** Study Population.

	Years									
	2014		2015		2016		2017		2018	
	
	n	%	n	%	n	%	n	%	n	%
	
***Gender:***										
**Men**	421	32.4	401	33.3	369	33.9	294	32.3	275	31.7
**Women**	878	67.6	803	66.7	721	66.1	616	67.7	592	68.3

***Age:***										
**20**–**40**	145	12.0	110	9.8	62	6.2	65	7.7	53	6.7
**41**–**60**	573	47.2	536	47.9	490	49.2	396	46.9	409	51.6
**61**–**80**	439	36.2	415	37.1	397	39.9	338	40.0	282	35.6
**>80**	56	4.6	58	5.2	46	4.6	46	5.4	48	6.1

***Relationship with cancer patient:***										
**Family member**	1210	90.9	1135	93.2	1039	93.9	889	95.2	818	93.7
**Relative**	88	6.6	46	3.8	48	4.3	30	3.2	39	4.5
**Friend**	6	0.5	6	0.5	5	0.5	4	0.4	1	0.1
**Other**	27	2.0	31	2.5	15	1.4	11	1.2	15	1.7

***Level of education:***										
**Early secondary school education**	415	32.5	383	33.0	364	34.2	302	34.1	269	32.1
**High school degree**	623	48.8	538	46.4	484	45.5	375	42.4	394	47.0
**Higher education degree**	238	18.7	239	20.6	215	20.2	208	23.5	175	20.9
